# Synthesis of Mesoporous CuO Hollow Sphere Nanozyme for Paper-Based Hydrogen Peroxide Sensor

**DOI:** 10.3390/bios11080258

**Published:** 2021-07-30

**Authors:** Dong Cheng, Jing Qin, Youyou Feng, Jing Wei

**Affiliations:** The Key Laboratory of Biomedical Information Engineering of Ministry of Education, Institute of Analytical Chemistry and Instrument for Life Science, School of Life Science and Technology, Xi’an Jiaotong University, Xi’an 710049, China; cd20190911@stu.xjtu.edu.cn (D.C.); soyoung@stu.xjtu.edu.cn (J.Q.); qq512182235@stu.xjtu.edu.cn (Y.F.)

**Keywords:** polyphenol, mesoporous metal oxide, hollow sphere, nanozyme, paper sensor

## Abstract

Point-of-care monitoring of hydrogen peroxide is important due to its wide usage in biomedicine, the household and industry. Herein, a paper sensor is developed for sensitive, visual and selective detection of H_2_O_2_ using a mesoporous metal oxide hollow sphere as a nanozyme. The mesoporous CuO hollow sphere is synthesized by direct decomposition of copper–polyphenol colloidal spheres. The obtained mesoporous CuO hollow sphere shows a large specific surface area (58.77 m^2^/g), pore volume (0.56 cm^3^/g), accessible mesopores (5.8 nm), a hollow structure and a uniform diameter (~100 nm). Furthermore, they are proven to show excellent peroxidase-like activities with *K*_m_ and *V*_max_ values of 120 mM and 1.396 × 10^−5^ M·s^−1^, respectively. Such mesoporous CuO hollow spheres are then loaded on the low-cost and disposable filter paper test strip. The obtained paper sensor can be effectively used for detection of H_2_O_2_ in the range of 2.4–150 μM. This work provides a new kind of paper sensor fabricated from a mesoporous metal oxide hollow sphere nanozyme. These sensors could be potentially used in bioanalysis, food security and environmental protection.

## 1. Introduction

Hydrogen peroxide (H_2_O_2_) is a strong oxidant and bleaching agent. It has been widely applied in biomedicine, the household and industry. H_2_O_2_ is also a reactive oxygen species (ROS), which play essential roles in many physiological and pathological processes [1,2,3,4]. Furthermore, H_2_O_2_ is linked to many human diseases, including cardiovascular disorders, diabetes, Parkinson’s disease, Alzheimer’s disease, Huntington’s disease, metabolic diseases and cancers [5,6]. Therefore, detection of H_2_O_2_ is very important for both academic and industrial purposes. The development of low-cost, simple, fast, sensitive and selective H_2_O_2_ sensors is imperative. At present, various sensors have been developed to detect hydrogen peroxide [7]. Generally, the detection methods mainly include electrochemical methods [8,9], chromatography [10], fluorescence [11,12], chemiluminescence [13,14], colorimetry [15,16,17] etc. However, most of the methods require the use of expensive and bulky instruments and equipment. As a result, there is an urgent need to develop simple, low-cost detection methods that do not require bulky instruments.

Paper sensors have been widely used in point-of-care detection due to their simplicity, low cost, visual detection, portability and minimal sample consumption [18,19,20]. It can be useful for qualitative and quantitative analyses with a range of analysis. At present, many paper-based sensing platforms have been developed using nanomaterials to enhance the detecting signals [21]. These nanomaterials showed efficient enzyme-like activity [18,22,23]. For example, Zhang et al. fabricated a paper sensor with mesoporous carbon loaded with Pd nanoparticles as a highly active peroxidase mimic for H_2_O_2_ detection [24]. Since the discovery of the Fe_3_O_4_ nanozyme in 2007 [25], metal oxide nanomaterials have been widely studied as efficient enzyme mimics. At present, CeO_2_ [26], NiO [27], MnO_2_ [28], V_2_O_5_ [29] and CuO [30], and others [31,32], have been found to show enzyme-like activities, such as peroxidase, catalase, oxidase and other activities. Ceria oxide and iron oxide are the most widely studied nanozymes [26,33]. Comparably, copper oxide is relatively less studied. CuO, a transition metal oxide with a narrow bandgap (~2.0 eV), has many excellent properties, such as low cost, easy mixing with polymers and relative stability in terms of both chemical and physical properties, high surface-to-volume ratio and easy preparation [34,35]. Therefore, CuO nanomaterials of various structures have been synthesized [36,37,38,39] and used for antibacterial, antioxidant and sensing purposes [40,41,42].

Mesoporous metal oxide hollow spheres exhibit a tunable pore size (2–50 nm), a large specific surface area, tailorable compositions, highly accessible pore channels and shortening mass transfer pathways. They have attracted broad applications in catalysis, sensors, energy conversion and storage [43,44]. When mesoporous metal oxide hollow spheres are used for nanozyme and paper-based sensors, they demonstrate several advantages. Firstly, mesoporous metal oxide hollow sphere nanozymes have a large specific surface area and a large number of accessible active sites [45,46]. Such active sites would facilitate the catalytic reaction on the surface of nanozyme and increase the sensitivity. Secondly, mesoporous metal oxide hollow sphere nanozymes show interconnected mesoporous channels, large pore sizes and a uniform diameter, which can favor mass transfer and thus enable fast detection [47,48]. Thirdly, mesoporous metal oxide hollow sphere nanozymes have large internal voids that can load other guests, such as enzymes, metal nanoparticles and reporter molecules [49,50]. Due to the flexibility of mesoporous structure and compositions, mesoporous metal oxide hollow sphere nanozymes have many applications in biosensors, antibacterial drug delivery and therapy. Until now, there has been no report on the synthesis of mesoporous CuO hollow sphere nanozymes for paper-based H_2_O_2_ sensors.

**Scheme 1:**
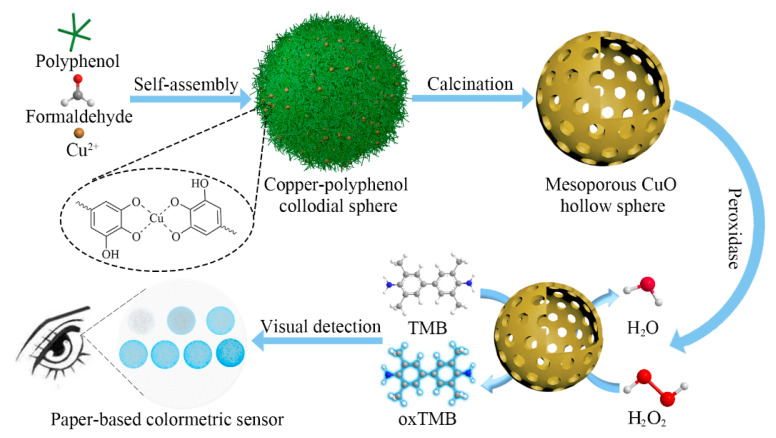
Schematic illustration of the synthesis of mesoporous CuO hollow sphere nanozymes and their application as a paper-based sensor for colorimetric detection of H_2_O_2_.

Herein, mesoporous CuO hollow sphere nanozymes are prepared for paper-based H_2_O_2_ sensors (Scheme 1). A sol–gel synthesis strategy is used to prepare copper–polyphenol coordination colloidal spheres using plant polyphenol (i.e., tannic acid, (TA)) as a polymerizable ligand, formaldehyde as a crosslinker and cupric ions as a metal source. After further thermal decomposition, mesoporous CuO hollow spheres are obtained. The obtained mesoporous CuO hollow spheres show a large specific surface area, large mesopore size, uniform particle size and excellent peroxidase activity. Such nanozymes are then used to fabricate low-cost, easy-to-use, portable and disposable paper sensors for the detection of H_2_O_2_.

## 2. Materials and Methods

### 2.1. Materials

Tannic acid (TA), Cu(NO_3_)_2_·3H_2_O, ethanol, hydrogen peroxide (30 wt%), 3,3′,5,5′-tetramethylbenzidine (TMB), L-proline, glycine, cysteine, alanine, glutamic acid, NaCl, KCl, MnCl_2_·4H_2_O, CaCl_2_, glucose and L-glutathione reduced were purchased from Macklin Biochemical Co., Ltd. Ammonia solution (25–28 wt%) and formaldehyde (37–40 wt%) were purchased from Tianjin Zhiyuan Chemical Co., Ltd. Pluronic^®^ F127 was purchased from Sigma-Aldrich. The qualitative filter paper was purchased from Whatman. All the reagents were used without further purification. Deionized water from a Milli-Q Plus system (Millipore) was used in all experiments.

### 2.2. Synthesis of Mesoporous CuO Hollow Sphere

The mesoporous CuO hollow sphere was synthesized by direct decomposition of metal–polyphenol colloidal spheres. Metal–polyphenol colloidal spheres were synthesized according to our previous reports with minor modifications [51,52]. The detailed synthesis procedure is shown in the Supplementary Materials. Mesoporous CuO hollow spheres were obtained by calcination of metal–polyphenol colloidal spheres at 350 °C for 2 h in the air with a ramping rate of 2 °/min. The metal–polyphenol colloidal spheres and their derived mesoporous CuO hollow spheres were denoted as Cu-TA and Cu-TA-350, respectively.

### 2.3. Mimic Peroxidase Activity of Mesoporous CuO Hollow Sphere

The mimic peroxidase properties of Cu-TA-350 were tested by dispersing 20 μL of Cu-TA-350 (1 mg/mL) in water solutions in the presence of 150 μL of PBS buffer (pH = 5.0) and 80 μL of TMB (10 mM) and H_2_O_2_ (10–600 mM) with a total volume of 1.0 mL. After the mixed solution was processed at room temperature for 5 min, the photographs and UV spectra of the mixtures were taken. The kinetic analysis of the peroxidase-like activity of Cu-TA-350 was investigated by monitoring absorbance after changing the concentration of H_2_O_2_. To analyze the reaction kinetic, the absorbance variation of the reaction solution was recorded in time scan mode at 652 nm. The kinetic parameters of the catalytic reaction were calculated based on the Michaelis–Menten function *v* = *V_max_* [S]/(*K_m_* + [S]), where *v* is the initial velocity, *V_max_* represents the maximal reaction velocity, [S] corresponds to the concentration of substrate, and *K_m_* is the Michaelis constant.

### 2.4. H_2_O_2_ Detection Using Mesoporous CuO Hollow Sphere

For H_2_O_2_ detection, Cu-TA-350 (20 μL, 1 mg/mL) was added into aqueous solution containing 150 μL of PBS buffer (0.5 mM, pH = 5.0), 80 μL of TMB (10 mM). After 5 min, H_2_O_2_ was added to the solution. The final concentration of H_2_O_2_ ranges was from 10 to 600 μM. After another 5 min, the absorbance was recorded with a UV–Vis spectrophotometer. The corresponding color changes of the reaction solution were photographed by a smartphone. To verify the selectivity of the Cu-TA-350 based colorimetry, common cations (K^+^, Na^+^, Ca^2+^, Mg^2+^ and Mn^2+^) and other substrates (ascorbic acid (AA), glucose (Glc), proline (Pro), glycine (Gly), glutamate (Glu) and alanine (Ala)) were similarly tested.

### 2.5. Fabrication of Paper-Based Sensor

For the fabrication of a paper-based sensor, filter paper with a pore size of 8 μM was immersed in Cu-TA-350 (1 mg/mL) solution for 5 min followed by drying in air. Then the filter paper was cut into pieces of 1 cm × 1 cm and stored at room temperature. For the detection of H_2_O_2_, 5 μL of TMB (10 mM) solution was dropped onto the paper piece and dried at room temperature for 30 s. Subsequently, 5 μL of samples with different concentrations of H_2_O_2_ were dropped on each corresponding paper piece. H_2_O_2_ was the substate of this catalytic reaction. Cu-TA-350 was used to oxidize the colorless substrate TMB into a blue product. After reacting for 5 min, the samples were rapidly photographed using a smartphone (iPhone 12). The camera was located at a constant height of 10 cm above the paper. Every test was under the same indoor conditions. The photograph of the paper was analyzed for the RGB value. The average of these individual color readings was calculated for each strip and was plotted as a function of different concentrations of H_2_O_2_ (10–150 μM). The selectivity of the sensor was evaluated by dropping different common cations (K^+^, Na^+^, Ca^2+^, Mg^2+^ and Mn^2+^) and other substrates, including ascorbic acid (AA), cysteine (Cys), glucose (Glc), proline (Pro), glycine (Gly), glutamate (Glu) and alanine (Ala), on the paper-based sensor. The concentrations of metal ions and glucose were 1 mM, while the concentrations of AA, GSH and amino acids were 100 μM. The repeatability of the paper-based sensor was evaluated by measuring the color of 9 batches of the paper-based sensor. The paper-based sensor was stored in sealed bags at 25 °C. The storage stability was assessed by measuring the color intensity of identical paper-based sensor in response to H_2_O_2_ (100 μM) at various time intervals.

## 3. Results

### 3.1. Synthesis and Characterization of Mesoporous CuO Hollow Spheres

Mesoporous CuO hollow spheres were synthesized via a modified sol–gel process (Scheme 1). Firstly, copper–polyphenol colloidal spheres were synthesized via a formaldehyde-assisted metal–ligand crosslinking strategy using tannic acid as a polymerizable ligand, cupric ions as a metal source and formaldehyde as a crosslinker in the alkaline condition. Secondly, mesoporous CuO hollow spheres were obtained using the copper–polyphenol colloidal spheres as a precursor via a direct thermal decomposition process. During the calcination process, the organic framework was decomposed into gaseous CO_2_ and H_2_O, which can induce the formation of the mesoporous framework. The inhomogeneous shrinkage during this calcination process induced the formation of the hollow structure. The metal–polyphenol colloidal spheres and their derived mesoporous CuO hollow sphere were denoted Cu-TA and Cu-TA-350, respectively.

**Figure 1:**
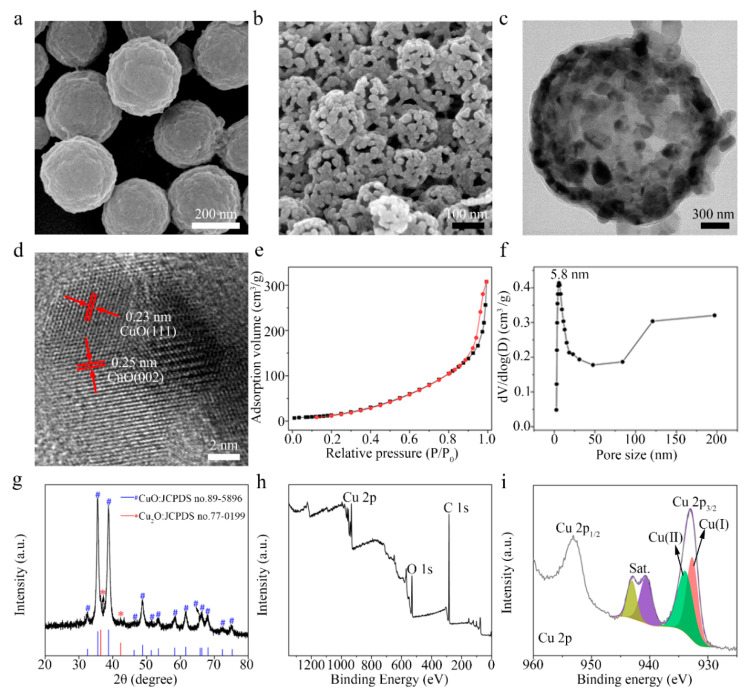
SEM images of (**a**) Cu-TA and (**b**) Cu-TA-350. (**c**) TEM and (**d**) high-resolution TEM images for Cu-TA-350. (**e**) N_2_ sorption isotherms and (**f**) corresponding pore size distributions for Cu-TA-350. (**g**) XRD patterns, (**h**) XPS survey spectra and (**i**) Cu 2p spectra for Cu-TA-350.

SEM image for Cu-TA revealed spherical morphology (Figure 1a). The average diameter was approximately 200 nm. After calcination, the obtained Cu-TA-350 had retained spherical structure (Figure 1b). The average diameter of Cu-TA-350 was around 100 nm. The sharp decrease in particle size was due to severe shrinkage during the thermal decomposition process. It should be noted that the sphere showed an obvious mesoporous framework. Transmission electron microscopy (TEM) images of Cu-TA-350 further confirmed the spherical structure with large voids (Figure 1c). The high-resolution TEM image of Cu-TA-350 showed a crystalline structure with a d-spacing of 0.23 and 0.25 nm, which could be assigned to the (111) and (002) planes of mesoporous CuO, respectively (Figure 1d). The selected area electron diffraction (SAED) pattern of Cu-TA-350 revealed a polycrystalline feature (Figure S1a). Furthermore, nitrogen sorption isotherms (Figure 1e) showed that Cu-TA-350 exhibited a typical IV-type desorption curve, indicating a mesoporous structure. The specific surface area and pore volume were 58.7 m^2^/g and 0.56 cm^3^/g, respectively. The pore size was 5.8 nm (Figure 1f).

X-ray diffraction (XRD) patterns of the Cu-TA-350 displayed distinct diffraction peaks, indicating a highly crystalline framework (Figure 1g). The diffraction peaks at 2θ values of 32.52°, 35.56°, 38.72°, 46.29°, 48.79°, 51.36°, 53.48°, 58.30°, 61.58°, 65.81°, 66.30°, 68.12° and 75.29° could be indexed to (110), (002), (111), (−112), (−202), (112), (020), (202), (−113), (022), (−311), (220), (311) and (−222) planes of crystalline CuO (JCPDS no.89-5896). Furthermore, XRD patterns also showed two weak diffraction peaks at 2θ values of 36.42° and 42.30°. They could be indexed to (111) and (200) planes of crystalline Cu_2_O (JCPDS no.77-0199). There is a small amount of Cu_2_O mixed in the CuO, which may be caused by the fact that part of the Cu is not safely oxidized during the calcination process [53]. X-ray photoelectron spectroscopy was used to study the surface properties and oxidation states of the Cu in the Cu-TA-350 (Figure 1h). The Cu 2p spectrum showed two peaks at binding energies (BEs) of 933 and 953.1 eV, which were ascribed to the Cu 2p_3/2_ and Cu 2p_1/2_ lines, respectively. The Cu 2p_3/2_ peak could be fitted to two peaks with BEs of 934.0 and 932.7 eV, corresponding to Cu(I) and Cu(II), respectively. Moreover, shakeup satellites were at 940.6 and 943.1 eV. These results demonstrated that the mesoporous CuO hollow sphere was successfully synthesized.

### 3.2. Peroxidase-Like Activity

**Figure 2:**
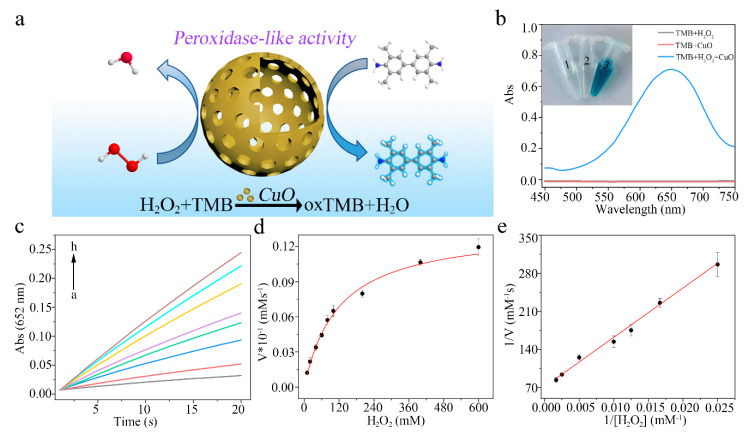
(**a**) Schematic detection of H_2_O_2_ using mesoporous CuO hollow sphere nanozymes. (**b**) UV–Vis absorption spectra of TMB + H_2_O_2_ (1), TMB + CuO (2) and TMB + H_2_O_2_ + CuO (3). Insets correspond to optical photographs. (**c**) Time-dependent absorbance at 652 nm with different concentrations of H_2_O_2_: (a) 10, (b) 20, (c) 40, (d) 80, (e) 100, (f) 300, (g) 400 and (h) 600 mM. (**d**) Steady-state kinetic analysis and (**e**) double-reciprocal plotting for Cu-TA-350 with H_2_O_2_ as the substrate.

To investigate the peroxidase-like activity of mesoporous CuO hollow spheres, a typical reaction of the catalytic oxidation of 3,3′,5,5′-tetramethylbenzidine (TMB) in the presence of H_2_O_2_ was adopted. As depicted in Figure 2a, Cu-TA-350 can catalyze the oxidation of TMB to form oxidized TMB (oxTMB). Mesoporous CuO hollow spheres can break the O-O bond of H_2_O_2_, producing two·OH, while TMB can be oxidized by ·OH to form oxTMB. The colorless mixture of TMB and H_2_O_2_ changed to a blue solution after adding Cu-TA-350. In contrast, negligible absorbance was observed in the presence of TMB and H_2_O_2_ (Figure 2b). It could be observed that Cu-TA-350 has enzymatic activity, while Cu^2+^ plays a role in enzymatic catalysis (Figure S2). These results successfully identified the peroxidase-like activity of Cu-TA-350 rather than the leached Cu^2+^. Meanwhile, the absorbance of the system at 652 nm noticeably increased as the reaction proceeded over 5 min (Figure S3a). The absorbance value increased gradually when more Cu-TA-350 was used. Therefore, Cu-TA-350 dispersed solution was selected for the following experiments. In addition, similar to the natural enzyme, the effects of the concentration of TMB, pH value and temperature on peroxidase-like activity of Cu-TA-350 were investigated. The absorbance gradually increased when the concentrations of TMB increased (Figure S3b). The peroxidase activity of Cu-TA-350 was best achieved at a pH value of 5.0 (Figure S3c) or at a temperature of 40 °C (Figure S3d). In the buffer solution with a pH of 5, Cu-TA-350 may show enhanced interaction with TMB molecules, promoting the catalytic oxidation of TMB with H_2_O_2_ [54].

To further evaluate the peroxidase-like activity of Cu-TA-350, the steady-state catalytic kinetics were investigated at room temperature in a reaction system containing Cu-TA-350, TMB and H_2_O_2_ of varied concentrations (10–600 mM) in PBS buffer solution. The time-dependent absorbance variation of the reaction solution was monitored in time scan mode at 652 nm using a UV–Vis spectrophotometer (Figure 2c). Typical Michaelis–Menten curves were also obtained by altering the concentration of H_2_O_2_ (Figure 2d). Then, *K_m_* and *V_max_* of the catalytic reaction by Cu-TA-350 were determined by the Lineweaver–Burk plot (Figure 2e). *K_m_* and *V_max_* values were calculated to be 12.67 × 10^−1^ M and 1.396 × 10^−5^ M s^−1^, respectively, which was comparable to other excellent nanozymes (Table S1).

### 3.3. H_2_O_2_ Detection

**Figure 3:**
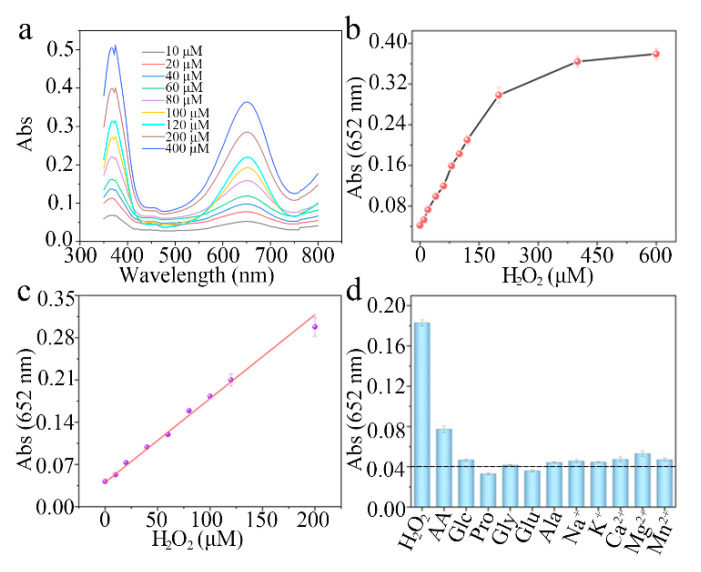
(**a**) UV–Vis absorbance spectra of Cu-TA-350 at different concentrations of H_2_O_2_ (10–400 μM). (**b**) Absorption spectra of Cu-TA-350 at different concentrations of H_2_O_2_. (**c**) The linear absorbance response in the H_2_O_2_ concentration ranged from 10 to 200 μM. (**d**) UV–Vis absorbance spectra of Cu-TA-350 solution in the presence of different H_2_O_2_, AA, GSH and amino acids (100 μM each). The concentrations of metal ions and glucose were 1 mM. The black line represents the value of the blank group.

TMB can be oxidized in the presence of H_2_O_2_ by the catalysis of Cu-TA-350. The oxidized TMB solution showed a blue color with strong absorption at 652 nm. The absorption was depended on the concentration of H_2_O_2_. It can be used as a colorimetric assay for H_2_O_2_ by monitoring the production of colored products at 652 nm by spectroscopy or visual observation. When the concentration of H_2_O_2_ varied from 10 to 600 μM, the intensity of absorbance peak gradually increased (Figure 3a,b). The optical photos of different concentrations of H_2_O_2_ (0–600 μM) reaction samples are shown in Figure S4. The linear relationship was in the range of 10–200 μM. The detection limit of H_2_O_2_, was 2.1 μM (Figure 3c). In addition, to test selectivity, control experiments were conducted using common metal ions and amino acids. The H_2_O_2_ group showed a high absorbance at 652 nm, and the color of TMB significantly changed (Figure 3d). These results indicated that Cu-TA-350 was expected to realize the detection of H_2_O_2_ in complex samples. According to the fact that H_2_O_2_ is used as a preservative agent in milk [55], the practicability of mesoporous Cu-TA-350 in the detection of H_2_O_2_ in commercial milk was performed. As shown in Table S2, there was no H_2_O_2_ detected as expected. When adding different concentrations of H_2_O_2_ to the samples, the recovery was in the range of 99.8–105.5%. All relative standard deviation (RSD) was below 3.5%, indicating that Cu-TA-350 was reliable and applicable to detect the H_2_O_2_ in complicated samples.

### 3.4. Paper-Based Sensor

**Figure 4:**
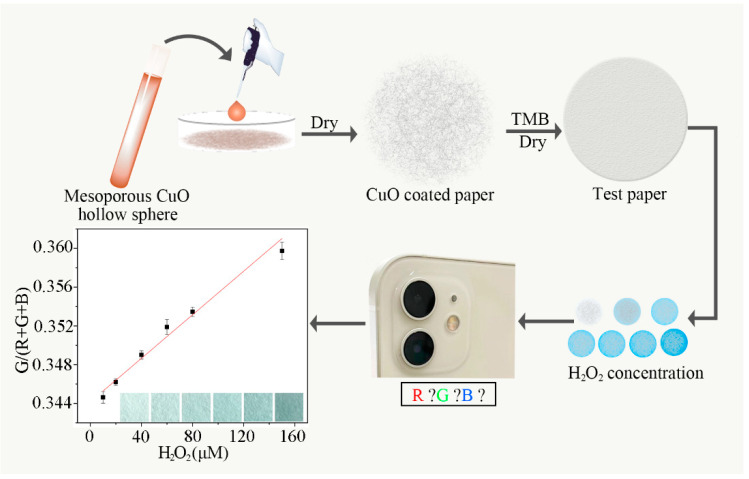
Schematic illustration of the fabrication of mesoporous metal oxide hollow sphere nanozyme-based paper as a colorimetric sensor for H_2_O_2_ detection. A smartphone was used to analyze the results.

The mesoporous CuO hollow spheres were then used to fabricate the paper-based chemical sensor for detection of H_2_O_2_ (Figure 4). The paper-based sensor was fabricated by deposition of CuO and TMB on the filter paper. Mesoporous CuO hollow spheres were well loaded onto the filter paper (Figure S5). The loading amount was around 0.17 mg/cm^2^ (Figure S6). When 5 μL of different concentrations of H_2_O_2_ was dropped on the paper sensor, the paper sensor showed a blue color. The color could be read by the camera of a smartphone (e.g., an iPhone 12). Finally, the complementary blue color was used for quantitative analysis of the concentration of H_2_O_2_. As a result, H_2_O_2_ can be quantified by the fitting relationship between the RGB ratio and H_2_O_2_ concentration. Figure 4 shows the linear relationship of the intensity of RGB to H_2_O_2_ concentration in the range of 10–150 μM and the G/(R + G + B) value of 0.344–0.366. The lowest detectable concentration of the paper-based visual sensor is approximately 2 μM. The concentration of hydrogen peroxide in the environment can reach 2.23 μM [56]. The concentration of hydrogen peroxide in the cells of the human body is from 50 to 100 µM [57]. The likely normal range of H_2_O_2_ in plasma is 1–5 μM. It may go up to as high at 50 μM in cases of inflammatory disease [58]. Compared with previously reported H_2_O_2_ sensors (Table S3), the proposed paper-based sensor has a desirable linear range and limit of detection.

**Figure 5:**
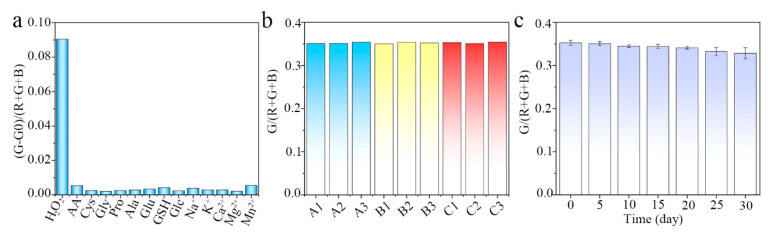
(**a**) Comparison of the sensing performance for various species H_2_O_2_, AA, Cys, Gly, Pro, Ala, Glu, GSH, Glc, Na^+^, K^+^, Ca^2+^, Mg^2+^ and Mn^2+^ using a paper-based sensor. The concentration of AA, GSH and amino acids was 100 μM. The concentration of metal ions and glucose was 1 mM. (**b**) Repeatability of the paper-based sensor for H_2_O_2_ detection by nine measurements from three batches (A, B and C). (**c**) Stability of the fabricated paper-based for H_2_O_2_ detection at room temperature.

To the best of our knowledge, the detection of H_2_O_2_ using a mesoporous CuO hollow sphere-based paper sensor has not yet been explored. Further study was also performed on the selectivity of the paper-based sensor for H_2_O_2_ detection. The paper-based sensor showed no obvious response in absence of H_2_O_2_ (Figure 5). This result demonstrated that the paper-based sensor had good selectivity of H_2_O_2_ (Figure 5a). Nine repeated experiments showed that the paper-based sensor had good repeatability (Figure 5b). Furthermore, the paper-based sensors can be stored for at least one month at 25 °C in a sealed bag (Figure 5c). These results demonstrated that the paper-based sensor showed excellent performance and could potentially be applied in practical applications.

## 4. Conclusions

In summary, a mesoporous CuO hollow sphere nanozyme is synthesized to fabricate a paper-based H_2_O_2_ sensor. The mesoporous CuO hollow sphere nanozyme is synthesized by direct thermal decomposition of metal–polyphenol coordination polymers. The obtained CuO nanozyme shows a high specific surface area, large pore size and hollow structure. Such features can effectively enhance the peroxidase activity of the CuO nanozyme. The paper-based sensor can be used for colorimetric detection of H_2_O_2_ in the range of 2.4–150 μM. Due to the hollow structure and mesoporous framework, such nanozymes could be used as a container to load other metal nanoparticles (e.g., Au) or natural enzymes, which would further expand the applications in the detection of other substances or biomedical therapy.

## Supplementary Materials

The following are available online at https://www.mdpi.com/article/10.3390/bios11080258/s1, Figure S1: Characterization of Cu-TA-350, Figure S2: Catalytic activity of leaching solution and mesoporous CuO hollow sphere, Figure S3: Effect of different conditions on the activity of Cu-TA-350 nanozyme, Figure S4: Optical photographs of different concentrations of H_2_O_2_ reaction samples, Figure S5: SEM images of paper substrate without and with the deposition of Cu-TA-350, Figure S6: TG curves for filter paper and filter paper loaded with Cu-TA-350, Table S1: Comparison of the peroxidase-like activity of different nanomaterials of H_2_O_2_, Table S2: Detection of H_2_O_2_ in commercial milk samples, Table S3: Comparison of various nanomaterial-based sensors for H_2_O_2_ detection.
