# Supplementary material for: Synthesis of Mesoporous CuO Hollow Sphere Nanozyme for Paper-Based Hydrogen Peroxide Sensor
Source: Biosensors (Basel). 2021 Jul 30;11(8):258. doi: 10.3390/bios11080258 (PMC8392683; doi:10.3390/bios11080258)
Supplement: Supplementary file 1 [file biosensors-11-00258-s001.zip › biosensors-1298004-supplementary.pdf]

biosensors

MDPI

Supplementary Information

Synthesis of Mesoporous CuO Hollow Sphere Nanozyme for Paper-based Hydrogen Peroxide Sensor

Synthesis of mesoporous CuO hollow sphere

Typically, F127 (0.2 g) was dissolved in the mixture of water (37 mL), ethanol (8 mL) and ammonia solution (0.35 mL, 25 wt%). TA (0.2 g in 8 mL of water) solution was then added to the above solution. Formaldehyde solution (3.8 mL, 3.7 wt%) was added to cross-link the polyphenol. After stirring for 24 hours, 2 mL of $Cu(NO_3)_2$ solution (containing 0.1 g of $Cu(NO_3)_2$) was added to the above solution. After another 24 h, the solution was transferred into an autoclave (100 mL) for hydrothermal treatment at 100 °C for 12 hours. The metal-polyphenol colloidal spheres were collected by centrifugation, washing and drying. Mesoporous CuO hollow sphere were obtained by calcination at 350 °C for 2 hours in air with a ramping rate of 2 °/min.

Characterization

Scanning electron microscopy (SEM) images were obtained on a Gemini SEM500. Transmission electron microscopy (TEM) images and selected area electron diffraction (SAED) patterns were taking using a JEM-F200. Nitrogen adsorption/desorption isotherms were measured with a Micromeritics Tristar 3020. X-ray diffraction (XRD) patterns were collected by a Bruker D8 Advance. X-ray photoelectron spectroscopy (XPS) spectra were recorded on a Kratos AXIS Ultra DLD system with Al Kα radiation as an X-ray source.

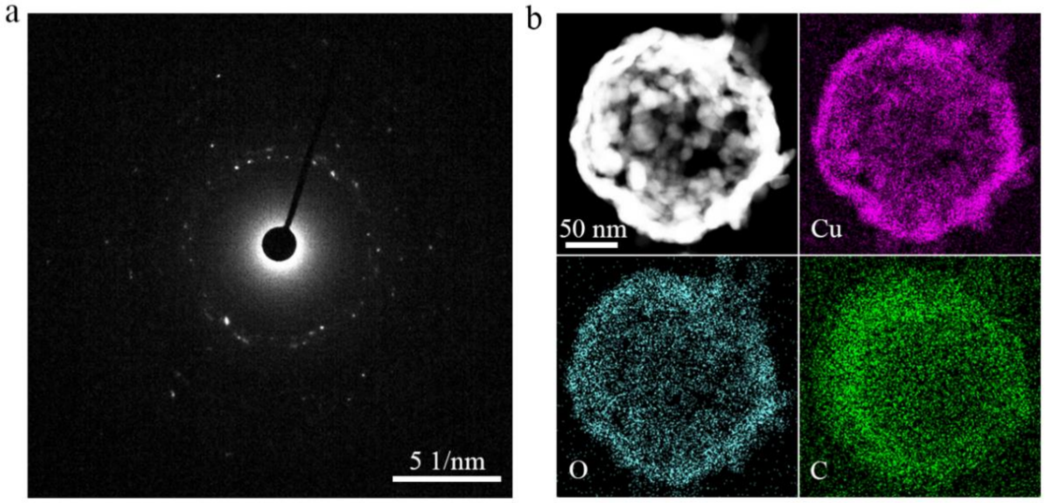

Figure S1. (a) Selected area electron diffraction (SAED) patterns. (b) STEM image and elemental mapping images of Cu-TA-350.

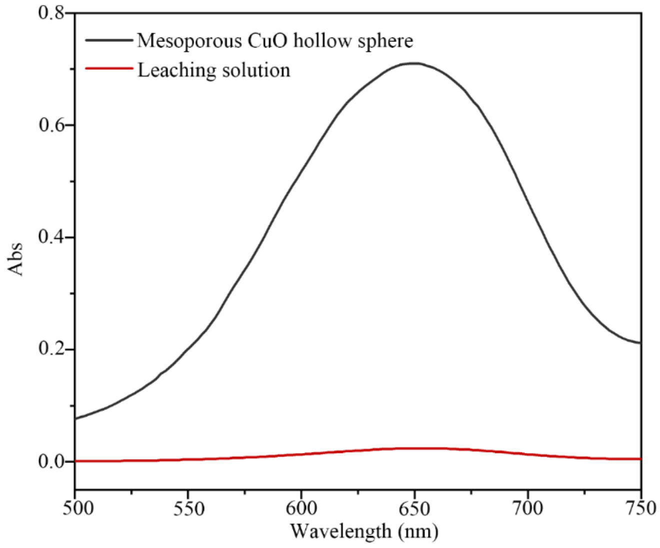

Figure S2. The catalytic activity of leaching solution and mesoporous CuO hollow sphere. The leaching solution was prepared by incubation of the Cu-TA-350 nanozyme in the buffer solution with a pH value of 5.0 for 30 min followed by removal of solid products.

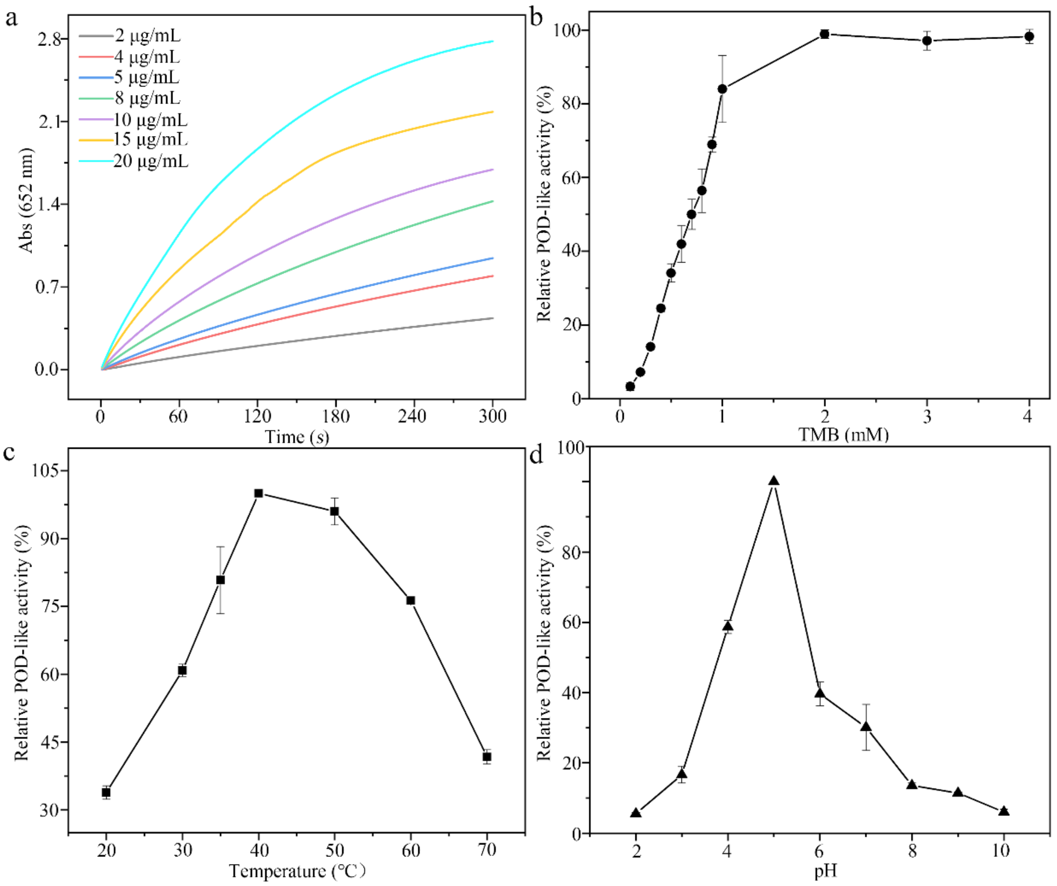

Figure S3. (a) Reaction-time curves of TMB colorimetric reactions in the present of different concentrations of Cu-TA-350 solution. (b-d) The relative POD-like activity at different conditions: (b) concentrations of TMB, (c) reaction temperature, (d) pH value in the reaction system.

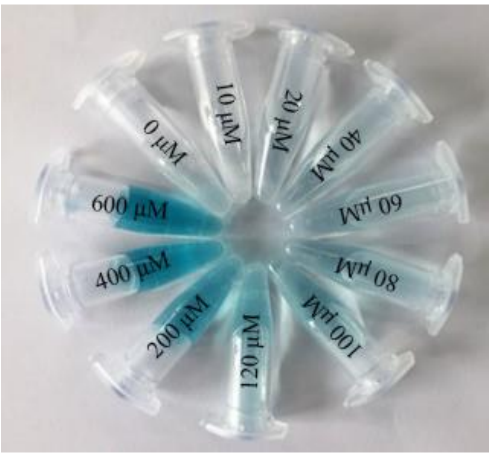

Figure S4. The optical photographs of different concentrations of H_2O_2 (0-600 μM) reaction samples.

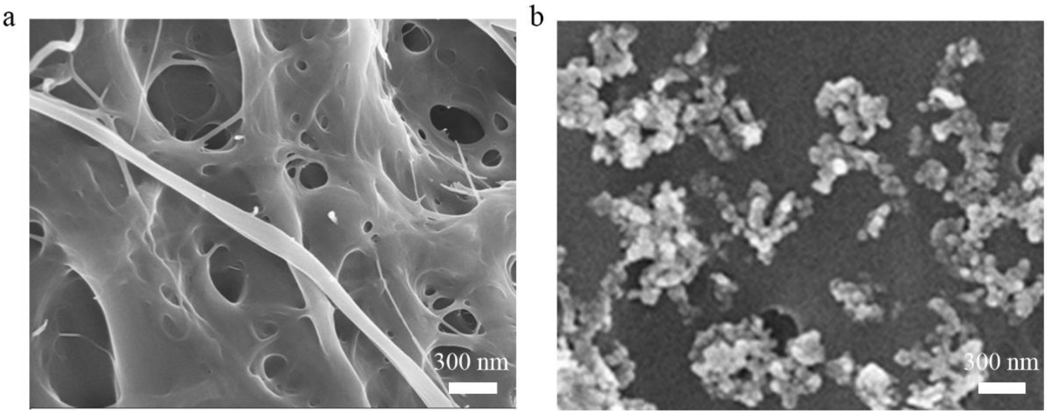

Figure S5. SEM images of paper substrate (a) without and (b) with the deposition of Cu-TA-350.

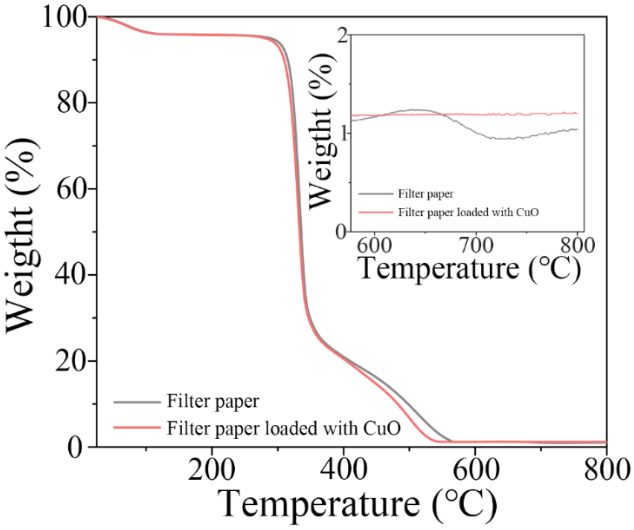

Figure S6. TG curves for filter paper and filter paper loaded with CuO-350 inset TG curves is a partial enlarged view.

Table S1. Comparison of the peroxidase-like activity of.different nanomaterials.

Catalyst	K_m (mM)	V_{max} (10^{-4} mM s^{-1})	refs
HRP	3.7	87.1	[1]
CuO	400	104.9	[2]
Fe_3O_4	154	79.8	[3]
Co_3O_4	140	121	[4]
Pure Cu_2O	118	13.6	[5]
Mesoporous CuO hollow sphere	120	0.14	This work

Table S2. Detection of H_2O_2 in commercial milk samples.

Sample	Added (μM)	Found (μM)	Recovery (%)	RSD (%)
Milk	0	/	/	/
	20	21.10	105.5	3.4
	50	49.88	99.8	0.83
	100	102.15	102.2	0.72

Table S3. Comparison of various nanomaterials-based sensors for H_2O_2 detection.

Materials	Liner range (μM)	Detection limit (μM)	Reference
CuO assembled on silicon nanowires	10-13180	1.6	[6]
CuO-g-C_3N_4	2-150	1.2	[7]
Co_3O_4 anchored to multiwalled carbon nanotubes	20-43	2.46	[8]
CuO/PANI hybrid nanofibers	5-9255	0.11	[9]
Graphene wrapped Cu_2O nanocubes	300-7800	20.8	[10]
Fe_3O_4@Cu@Cu_2O	4000-50000	2000	[11]
N-G-Fe_3O_4	0-10000	17.1	[12]
Cu_2O-rGO	30-12800	21.7	[13]
copper sulfide-decorated Ca-montmorillonite	30-200	24.7	[14]
$Cu_2(OH)_3Cl$-CeO_2	20-50	10	[15]
CuO/Cu	2-19.4	2.0	[16]
V_2O_5-CeO_2	20-180	3.0	[17]
CuO nanosheets on copper foil	10-20,000	10	[18]
Mesoporous CuO hollow sphere	10-150	2.4	This work

References

1. Dutta, A.K.; Maji, S.K.; Mondal, A.; Karmakar, B.; Biswas, P.; Adhikary, B. Iron selenide thin film: Peroxidase-like behavior, glucose detection and amperometric sensing of hydrogen peroxide. *Sens. Actuators B Chem.* **2012**, *173*, 724–731.

2. Nagvenkar, A.P.; Gedanken, A. $Cu_{0.89}Zn_{0.11}O$, A New Peroxidase-Mimicking Nanozyme with High Sensitivity for Glucose and Antioxidant Detection. *ACS Appl. Mater. Interfaces* **2016**, *8*, 22301–22308.

3. Gao, L.Z.; Zhuang, J.; Nie, L.; Zhang, J.B.; Zhang, Y.; Gu, N.; Wang, T.H.; Feng, J.; Yang, D.L.; Perrett, S.; et al. Intrinsic peroxidase-like activity of ferromagnetic nanoparticles. *Nat. Nanotechnol.* **2007**, *2*, 577–583.

4. Mu, J.S.; Wang, Y.; Zhao, M.; Zhang, L. Intrinsic peroxidase-like activity and catalase-like activity of Co_3O_4 nanoparticles. *Chem. Comm.* **2012**, *48*, 2540–2542.

5. Kuo, M.Y.; Hsiao, C.F.; Chiu, Y.H.; Lai, T.H.; Fang, M.J.; Wu, J.Y.; Chen, J.W.; Wu, C.L.; Wei, K.H.; Lin, H.C.; et al. Au@Cu_2O core@shell nanocrystals as dual-functional catalysts for sustainable environmental applications. *Appl. Catal. B* **2019**, *242*, 499–506.

6. Huang, J.F.; Zhu, Y.H.; Zhong, H.; Yang, X.L.; Li, C.Z. Dispersed CuO Nanoparticles on a Silicon Nanowire for Improved Performance of Nonenzymatic H_2O_2 Detection. *ACS Appl. Mater. Interfaces* **2014**, *6*, 7055–7062.

7. Zhu, J.L.; Nie, W.; Wang, Q.; Li, J.W.; Li, H.; Wen, W.; Bao, T.; Xiong, H.Y.; Zhang, X.H.; Wang, S.F. In situ growth of copper oxide-graphite carbon nitride nanocomposites with peroxidase-mimicking activity for electrocatalytic and colorimetric detection of hydrogen peroxide. *Carbon* **2018**, *129*, 29–37.

8. Heli, H.; Pishahang, J. Cobalt oxide nanoparticles anchored to multiwalled carbon nanotubes: Synthesis and application for enhanced electrocatalytic reaction and highly sensitive nonenzymatic detection of hydrogen peroxide. *Electrochim. Acta.* **2014**, *123*, 518–526.

9. Liu, T.J.; Guo, Y.Q.; Zhang, Z.F.; Miao, Z.C.; Zhang, X.Y.; Su, Z.Q. Fabrication of hollow CuO/PANI hybrid nanofibers for non-enzymatic electrochemical detection of H_2O_2 and glucose. *Sens. Actuators B Chem.* **2019**, *286*, 370–376.

10. Liu, M.M.; Liu, R.; Chen, W. Graphene wrapped Cu_2O nanocubes: Non-enzymatic electrochemical sensors for the detection of glucose and hydrogen peroxide with enhanced stability. *Biosens. Bioelectron.* **2013**, *45*, 206–212.

11. Wang, Z.H.; Chen, M.; Shu, J.X.; Li, Y. One-step solvothermal synthesis of Fe_3O_4@Cu@Cu_2O nanocomposite as magnetically recyclable mimetic peroxidase. *J. Alloys Compd.* **2016**, *682*, 432–440.

12. Zhang, W.J.; Chen, C.P.; Yang, D.X.; Dong, G.X.; Jia, S.J.; Zhao, B.X.; Yan, L.; Yao, Q.Q.; Sunna, A.; Liu, Y. Optical Biosensors Based on Nitrogen-Doped Graphene Functionalized with Magnetic Nanoparticles. *Adv. Mater. Interfaces.* **2016**, *3*, 1600590.

13. Xu, F.G.; Deng, M.; Li, G.Y.; Chen, S.H.; Wang, L. Electrochemical behavior of cuprous oxide-reduced graphene oxide nanocomposites and their application in nonenzymatic hydrogen peroxide sensing. *Electrochim. Acta.* **2013**, *88*, 59–65.

14. Zhang, L.Y.; Chen, M.X.; Jiang, Y.L.; Chen, M.M.; Ding, Y.A.; Liu, Q.Y. A facile preparation of montmorillonite-supported copper sulfide nanocomposites and their application in the detection of H_2O_2. *Sens. Actuators B Chem.* **2017**, *239*, 28–35.

15. Wang, N.; Sun, J.C.; Chen, L.J.; Fan, H.; Ai, S.Y. A $Cu_2(OH)_3Cl$-CeO_2 nanocomposite with peroxidase-like activity, and its application to the determination of hydrogen peroxide, glucose and cholesterol. *Microchim. Acta.* **2015**, *182*, 1733–1738.

16. Yuan, R.M.; Li, H.J.; Yin, X.M.; Zhang, L.L.; Lu, J.H. Stable controlled growth of 3D CuO/Cu nanoflowers by surfactant-free method for non-enzymatic hydrogen peroxide detection. *J Mar Sci Technol* **2018**, *34*, 1692–1698.

17. Luo, X.; Shu, H.; Wan, Q.J.; Wang, Z.H.; Yang, N.J. Biosensing Applications of V_2O_5-CeO_2 Mesoporous Silica. *Electroanalysis* **2015**, *27*, 2194–2200.

18. Hsu, Y.K.; Chen, Y.C.; Lin, Y.G. Spontaneous formation of CuO nanosheets on Cu foil for H_2O_2 detection. *Appl. Surf. Sci.* **2015**, 354, 85–89.
